# Health on the frontlines: Morbidity patterns and influencing factors among healthcare workers in southern India

**DOI:** 10.6026/973206300221794

**Published:** 2026-03-31

**Authors:** B. Venkatashiva Reddy, Vishnu Rajan, Ramireddy Gari Likhitha, Rajeev Aravindakshan

**Affiliations:** 1 Department of Community and Family Medicine, All India Institute of Medical Sciences, Mangalagiri, Andhra Pradesh, India; 2 Department of Community Medicine, MES Academy of Medical Sciences, Perinthalmanna, Kerala, India

**Keywords:** Frontline healthcare workers, occupational health, morbidity patterns, mixed-methods study, health-seeking behaviour

## Abstract

Frontline healthcare workers in India face heavy workloads, occupational hazards and limited access to occupational health services. This mixed-methods study conducted in a tertiary care institution and its rural primary health centre in southern India assessed morbidity patterns among 102 workers using quantitative surveys and qualitative interviews. Musculoskeletal disorders were most common (49%), followed by respiratory illnesses (18.6%) and mental health problems (7.8%), with nearly half anaemic and 38.2% overweight or obese. Participants reported physical strain, administrative burden, poor ergonomic support and barriers to healthcare access. Thus, we show a substantial morbidity burden and the need for integrated occupational health services, regular screening and supportive workplace interventions.

## Background:

A well-functioning health system highly depends on the workforce involved in it. Human resources are one of the six building blocks of health [1]. Yet, the very individuals who form the backbone of this system often face significant health challenges themselves [2]. Effective workforce coordination is essential for universal health coverage and managing shocks such as COVID-19. Frontline health workers such as Accredited Social Health Activists (ASHAs), Auxiliary Nurse Midwives (ANM), etc., form the foundation of the public health system. They act as the first point of contact for communities and ensuring that essential services reach even the most remote populations. Accredited Social Health Activists' (ASHAs) work involves extensive travel by foot, exposure to harsh weather conditions and long working hours [3]. Moreover, the emotional toll of dealing with health crises and community health challenges can contribute to mental health issues such as stress and anxiety [3, 4]. The Auxiliary Nurse Midwives (ANM) is tasked with providing essential maternal and child health services, yet they too face significant health challenges [3]. The chronic fatigue and burnout rates are increasing among these frontline workforces in India [4, 5-6]. Across these groups, common issues include heavy workloads, occupational hazards, poor ergonomic support and limited access to occupational health services [7]. The World Health Organization emphasizes that strengthening the health workforce requires both expansion and improved working conditions and health of existing workers [8]. Therefore, it is of interest to describe the morbidity profile of frontline healthcare workers in two healthcare settings of southern India and explore the occupational, environmental and socio-cultural factors contributing to their health issues.

## Methodology:

## Study design:

This study employed a mixed-methods research design, integrating quantitative and qualitative approaches to provide a comprehensive understanding of the health issues faced by ASHA, ANM, AWW and Nursing Orderlies and housekeeping staff in India. The quantitative component consisted of a cross-sectional survey that assessed the prevalence and types of health problems experienced by these groups. The qualitative component included in-depth interviews and focus group discussions, which explored the underlying factors contributing to these health issues as well as the lived experiences of the workers.

## Study population and setting:

The target population for this study includes ASHA, ANM, Nursing Orderlies and housekeeping staff working in a tertiary care institution and its rural primary health centre situated in Guntur District Andhra Pradesh, India. Participants were included in the study if they had a minimum of six months of continuous service in their roles and were between 18 and 60 years of age, ensuring adequate occupational exposure and representation of the active workforce. Workers on long-term leave or absent during the study period were excluded, along with individuals who had pre-existing chronic conditions unrelated to occupational exposure (e.g., congenital disorders or advanced non- occupational diseases) that could bias the morbidity assessment.

## Sample size and sampling strategy:

Assuming a confidence level of 95% (Z = 1.96), absolute precision of 7% and an estimated prevalence of high stress of 14% [6], the calculated sample size was approximately 102, with 25-26 individuals from each group. For the qualitative component, purposive sampling was used. Approximately eight in-depth interviews (two per group) and four focus group discussions (one per group) were conducted. A stratified random sampling technique with a sampling interval of four was employed. Written informed consent was obtained prior to data collection. Ethical approval was granted by the institutional ethics committee (AIIMS/MG/IEC/2025-26/313, dated 20 May 2025).

## Data analysis:

Quantitative data were analyzed using statistical software R version 4.4.1 (The R Foundation, Vienna, Austria). Descriptive statistics were used to summarize demographic characteristics and the prevalence of health issues. Chi-square tests and t-tests were performed to identify differences in health outcomes between groups and across demographic variables. Qualitative data were analyzed using thematic analysis, which involved identifying, interpreting and reporting recurring patterns within the dataset. The process began with familiarization, during which transcripts were read and re-read to develop an in-depth understanding of the content. This was followed by systematic coding of meaningful data segments across all transcripts. The codes were then organized into potential themes and the emerging themes were reviewed and refined to ensure that they accurately represented the data and aligned with the study objectives. Once finalized, each theme was clearly defined and named to reflect its central idea. Direct quotations from participants were incorporated to support these themes and provide a detailed and authentic account of the health issues and lived experiences of the target groups.

## Ethical considerations:

Ethical approval was obtained from the institutional review committee and written informed consent was obtained from all participants. Confidentiality and anonymity were maintained, with data stored securely and identifiers removed. Universal precautions were followed and risks were clearly explained. Laboratory procedures adhered to quality standards and biomedical waste was disposed of according to regulations. Relevant HMIS-based findings were shared with participants.

## Results and Discussion:

A total of 102 participants were included in the study. The median age of the participants was 40 years (IQR: 34-48). The study population was predominantly female, with 99 participants (97.1%). The largest proportion of participants had completed secondary education (40, 39.2%), followed by higher secondary education (27, 26.5%). Graduates and those with primary education each constituted 11.8% of the sample, while postgraduates accounted for 6.9%. A small proportion of participants (3.9%) reported having no formal education. The Accredited Social Health Activists (ASHA) formed the largest group (26, 25.5%), followed by housekeeping staff (24.5%). Auxiliary Nurse Midwives (ANM) and Anganwadi Workers (AWW) each constituted 13.7% of the participants. The median duration of working experience in the current institution was 9 months (IQR: 5-18) (Table 1 - see PDF). Health status and comorbidity details work characteristics and health- seeking behaviour of study participants are given in (Table 2 - see PDF). Nearly one-fifth of the participants reported a past history of diabetes mellitus (18, 17.6%), while hypertension was reported by 8 (7.8%) and thyroid disorders by 11 (10.8%). Other comorbid conditions were reported by 15 participants (14.7%). Within the preceding one-year, musculoskeletal illnesses were the most commonly reported health problem (50, 49.0%), followed by other work-related health issues (38, 37.3%). Respiratory illnesses were reported by 19 participants (18.6%), while mental health-related illnesses were reported by 8 (7.8%). Less than half of the participants reported having sought medical treatment for their health problems (48, 47.1%). At the time of the survey, 55 participants (53.9%) were availing some form of treatment, while treatment-seeking information was missing for 17 participants (16.7%). The median weekly working hours were 48 hours (IQR: 42-51). Exposure to hazardous materials at the workplace was reported by 27 participants (26.5%). Access to protective gear was reported as always available by 45 participants (44.1%), sometimes available by 26 (25.5%) and never available by 19 (18.6%), while 12 participants (11.8%) did not report on this aspect. Management support for health and safety was perceived as high by a considerable proportion of participants. More than half of the participants reported engaging in daily physical activity (63, 61.8%), while 26 (25.5%) reported rarely engaging in physical activity.

The workload was perceived as always manageable by the majority of participants (77, 75.5%). However, regular health check-ups were infrequent, with 61 participants (59.8%) reporting that they rarely underwent health check-ups. Most participants reported easy access to healthcare services (92, 90.2%) and 41 participants (40.2%) reported having a regular healthcare provider. Preventive health measures were used sometimes by 65 participants (63.7%), while 23 (22.5%) reported always using such measures. A specific health-related diet was followed by 27 participants (26.5%). Work-related stress was reported as rare by 55 participants (53.9%), while daily stress was reported by 15 (14.7%). Self-rated overall health was reported as fair by 45 participants (44.1%) and good by 44 (43.1%), with only 2 participants (2.0%) rating their health as poor. A majority of participants expressed a high willingness to participate in workplace health programs, with 71 (69.6%) reporting that they were very likely to participate. Nutrition education was the most preferred type of health program (52, 51.0%), followed by exercise-based programs (31, 30.4%) (Table 3 - see PDF). The anthropometric and biochemical markers of study participants are given in (Table 4 - see PDF). A majority of the participants were either obese class I (37, 36.3%) or obese class II (39, 38.2%). Anaemia was present in nearly half of the study population, with mild anaemia observed in 23 participants (22.5%), moderate anaemia in 23 (22.5%) and severe anaemia in 3 (2.9%). Mean haematocrit was 36.2 ± 4.5% and the mean red blood cell count was 4.57 ± 0.44 x 10^12^/L. Red cell indices showed a mean corpuscular volume of 79 ± 10 fL and a mean corpuscular haemoglobin concentration of 32.47 ± 1.22 g/dL. The mean total leucocyte count was 7.65 ± 1.56 x10^9^/L. Vitamin B12 deficiency was observed in 16 participants (15.7%), while vitamin D_3_ deficiency was present in 28 (27.5%). Thyroid function assessment showed elevated thyroid-stimulating hormone levels in 25 participants (24.5%) and reduced levels in 3 (2.9%). Dyslipidaemia was common, with elevated total cholesterol levels observed in 24 participants (23.5%) and high triglyceride levels in 24 (23.5%). Low high-density lipoprotein cholesterol levels were noted in more than half of the participants (52, 51.0%), while elevated low-density lipoprotein cholesterol levels were observed in 59 (57.8%) (Table 5 - see PDF). Liver and renal function parameters were largely within normal limits. Elevated total bilirubin levels were observed in 3 participants (2.9%), while serum creatinine was elevated in 1 participant (1.0%) (Table 6 - see PDF). Screening for infectious diseases showed that 2 participants (2.0%) tested positive for hepatitis B virus by RT-PCR, while HIV antibodies were detected in 1 participant (1.0%). No participant tested positive for hepatitis C virus (Table 7 - see PDF). Table 8 (see PDF) shows the distribution of clinical and biochemical characteristics of health-care workers by designation. A high prevalence of overweight and obesity was observed across all designations, with 73% of ANMs, 80% of ASHAs, 54% of AWHs, 71% of AWWs and 80% of housekeeping staff classified as overweight or obese (p=0.90). Anemia prevalence varied significantly by designation (p=0.049), being highest among ANMs (65%) and ASHAs (54%) and lowest among AWHs (18%). Consistently, mean hemoglobin levels differed significantly across groups (p=0.040). Vitamin D deficiency was common across all designations-27% in ANMs, 23% in ASHAs, 9.1% in AWHs, 36% in AWWs and 36% in housekeeping staff (p=0.50). Lipid profile parameters were comparable across groups. Among liver enzymes, AST levels showed a significant inter-group difference (p=0.032), while ALT and alkaline phosphatase did not. Qualitative results of in-depth interviews and focused group discussion are summarised in (Table 9 & 10 - see PDF) respectively. This mixed-methods study highlights a significant burden of physical, metabolic and occupational health problems among frontline health workers in southern India. The findings underscore the need to reframe workforce strengthening through a health and safety lens. A striking finding was the high prevalence of overweight and obesity, with nearly three-quarters of participants classified as obese class I or II. Similar trends have been reported among healthcare workers globally, where long working hours, shift work, irregular meals and limited time for physical activity contribute to unhealthy dietary behaviors and weight gain [9, 10]. Obesity in this group is particularly concerning because it coexists with diabetes mellitus and hypertension, both of which were present in a notable proportion of participants. This clustering of metabolic risk factors suggests an emerging cardiovascular risk profile among frontline workers, despite their role in health promotion. Anaemia remained a significant problem, affecting almost half of the participants. Mild and moderate anaemia accounted for most cases, consistent with earlier studies among ASHAs and Anganwadi workers [10, 11]. The persistence of anaemia among health workers reflects broader gendered and occupational inequities, including nutritional compromise, high workload and limited self-care. The presence of vitamin B12 and vitamin D_3_ deficiencies further supports the chronic nutritional inadequacy among the workers. These deficiencies have been reported among female health workers and are often linked to dietary patterns, reduced sun exposure and competing domestic responsibilities. Musculoskeletal disorders were the most commonly reported morbidity in the preceding year. Nearly half of the participants reported body pain, joint pain, or related symptoms. Qualitative findings strongly reinforced this observation. Studies indicate that between 43% and 78% of hospital personnel are affected by musculoskeletal disorders [12, 13]. Nearly half of the participants reported body pain, joint pain, or related symptoms. Qualitative findings strongly reinforced this observation. Workers frequently described persistent pain as an expected part of their job. Prolonged walking, repetitive movements, manual handling and lack of ergonomic support were commonly cited. Similar patterns have been documented among ASHAs, ANMs and hospital support staff [14, 15].

The normalization of pain and continued work despite symptoms may delay care seeking and contribute to chronic disability over time. Administrative and digital workload emerged as a major source of stress. Participants repeatedly referred to app-based reporting, repeated data entry and poor mobile functionality. These tasks were perceived as mentally taxing and time-consuming. While digital health systems are intended to improve efficiency and accountability, inadequate infrastructure and insufficient training can increase stress for frontline workers [16]. The findings suggest that digital interventions need to be redesigned with greater attention to usability and field realities. Mental health morbidity was reported by a relatively small proportion of participants. However, qualitative data revealed frequent experiences of stress, tension and emotional exhaustion. This discrepancy likely reflects under-recognition and under-reporting of mental health problems. Stigma, lack of formal diagnosis and limited access to mental health services are known barriers among health workers in India [16]. The presence of daily work-related stress among participants is concerning, given its association with burnout and reduced work performance [17, 3]. Health-seeking behaviour patterns revealed important gaps. Less than half of the participants had sought medical care for their health problems. Regular health check-ups were uncommon. Qualitative narratives pointed to lack of health insurance, restricted consultation timings and difficulties accessing timely care, even within the same health system. Similar findings have been reported in other studies, where health workers prioritize patient care over their own health and face structural barriers to accessing services [17, 3]. The expressed demand for periodic health check-ups and unified insurance coverage highlights unmet occupational health needs. Despite these challenges, several enabling factors were identified. Many participants perceived their workload as manageable and described supportive supervisors and strong peer networks. Family support was also frequently mentioned as a factor enabling continued work. Such social support has been shown to buffer occupational stress and enhance resilience among health workers [17]. Importantly, a large majority expressed willingness to participate in workplace health programs. Nutrition education and exercise-based interventions were the most preferred options, suggesting openness to preventive strategies. Biochemical findings indicated that liver and renal function were largely within normal limits. However, dyslipidaemia was common. More than half of the participants had elevated low-density lipoprotein cholesterol or low high-density lipoprotein cholesterol. Similar lipid abnormalities have been reported among healthcare workers and are often linked to stress, physical inactivity, the qualitative findings add depth to the quantitative results. The systemic neglect of self-care among hospital staff is reflected in objective health declines, with studies showing that female healthcare workers, in particular, experienced significant increases in BMI and blood pressure following the onset of the COVID-19 pandemic [18]. Participants described long-standing illnesses, episodic symptoms that interfered with work and coping strategies such as self-medication, rest, yoga and dietary modifications. They also highlighted system-level gaps, including lack of insurance, poor access to care during emergencies and inadequate transport facilities for night duties. These insights illustrate how health problems intersect with work organization and health system design. This study has important strengths. The mixed-methods approach allowed triangulation of prevalence data with lived experiences. Inclusion of nursing orderlies and housekeeping staff, which are often excluded from workforce research, strengthens the equity focus of the study. However, the findings should be interpreted with caution. The cross-sectional design limits causal inference. Self-reported morbidity may underestimate conditions such as mental illness. The study was conducted in a single district, which may limit generalizability.

## Conclusion:

The frontline health and care workers experience a substantial burden of metabolic, musculoskeletal and occupational health problems. These issues are closely linked to workload, administrative demands, nutritional vulnerabilities and gaps in worker protection. Strengthening the health workforce requires moving beyond recruitment and training. Protecting the health of health workers is fundamental to building resilient health systems and sustaining progress toward universal health coverage.
